# Inclusive leadership and subordinates’ career calling: roles of belongingness and organization-based self-esteem

**DOI:** 10.3389/fpsyg.2025.1415426

**Published:** 2025-03-20

**Authors:** Guangya Ma, Longmei Wang, Siwei Sun, Lei Lu

**Affiliations:** ^1^School of Foreign Studies, Yiwu Industrial and Commercial College, Jinhua, China; ^2^School of Economics and Management, Zhongshan Polytechnic, Zhongshan, China; ^3^Department of Management and International Business, Business School, The University of Auckland, Auckland, New Zealand; ^4^School of Psychological and Cognitive Sciences, Beijing Key Laboratory of Behavior and Mental Health, Peking University, Beijing, China

**Keywords:** inclusive leadership, career calling, belongingness, organization-based self-esteem, need-to-belong theory

## Abstract

**Introduction:**

While the concept of career calling has garnered attention for its role in inspiring employees’ sense of purpose and engagement, the literature on its developmental aspects, especially the influence of leadership styles, has not been fully paid attention to. Furthermore, the association between leadership styles and career calling still needs further exploration. Therefore, this study narrows this gap through testing the mediating role of belongingness in the relationship between inclusive leadership and career calling and its variation across different levels of organization-based self-esteem. Drawing upon the Need-to-Belong Theory, we propose a moderated mediation framework to elucidate the relationship and its variations between inclusive leadership and career calling among employees.

**Methods:**

Data was collected from 337 employees across various industries in Guangdong, Zhejiang, and Beijing, China, using a two-wave lagged questionnaire. We used Inclusive Leadership-9, Belongingness-12, Organization-Based Self-Esteem-10 and Career Calling-12 measurement variables. Later, we used SPSS and PROCESS to verify five hypotheses.

**Results:**

Statistical testing revealed that (1) there is a positive relationship between inclusive leadership and career calling. (2) Belongingness plays a mediating role in the relationship between inclusive leadership and career calling. (3) Organization-based self-esteem moderates the association between inclusive leadership and career calling, both directly and indirectly through belongingness.

**Conclusion:**

This study provides insights into the conversation about inclusive leadership and career calling, revealing a deeper understanding of the associations between leadership styles and employees’ vocational fulfillment, and suggesting practical implications for encouraging an inclusive work environment that supports career development.

## 1 Introduction

More and more individuals are working to pursue work meaningfulness, and find and achieve a career calling (Berg et al., 2010). Coincidentally with this reality, there has been a wave of research career calling in the field of management (DiRenzo et al., 2022; Dobrow et al., 2023; Ran et al., 2023; Thompson and Bunderson, 2019). Defined as strong enthusiasm and meaningful passion toward work perceived by individuals (Dobrow and Tosti-Kharas, 2011), career calling has attracted scholars’ attention and been verified as a predictor of individual and organizational aspects (Zhang and Jin, 2019), such as job crafting (Chang et al., 2021), task performance (Vianello et al., 2022), and job satisfaction (Huang et al., 2022). Given the positive role of career calling in managing employees and organizational development, scholars have devoted themselves to inspecting influential factors of career calling, including leadership (Zhang and Jin, 2019), personality (Wen et al., 2022), and organizational context (Chen et al., 2018). Despite the acknowledged importance of career calling in enhancing employee engagement and organizational growth, the exploration of factors influencing career calling (Dobrow et al., 2023; Thompson and Bunderson, 2019), particularly the association with leadership, remains limited.

As employees’ actions and attitudes will be deeply influenced by leaders (Choi et al., 2015), exploring the relationship between leadership and subordinates’ career calling is necessary. Past literature has tried to explore the effects of different types of leadership on career calling, such as empowering leadership (Zhang and Jin, 2019), spiritual leadership (Wu and Lee, 2020), and ethical leadership (Zhang and Jiang, 2020). Nevertheless, among different types of leadership, the relationship and the mechanism through which inclusive leadership is linked with career calling have not been addressed in the conversation. At present, many corporate tasks are completed in the form of teams, and employees from different departments form temporary teams. They not only have belongingness to the temporary teams, but also maintain their individual uniqueness. While a number of existing positive leadership methods have been identified as able to facilitate the completion of work tasks in temporary teams, none of them adequately address these basic needs of group members, namely the value of belongingness and uniqueness (Randel et al., 2018; Korkmaz et al., 2022). Inclusive leadership has the potential to be effective for both different work groups and the same work group (Brewer, 1991; Shore et al., 2011). Inclusive leadership is distinct from other leadership paradigms due to its emphasis on fostering an environment that values and embraces all group members, no matter what their backgrounds are or what perspectives they stand for (Hollander, 2009). Unlike other leadership styles that may focus primarily on performance outcomes or individual leader characteristics, inclusive leadership is characterized by behaviors that promote diversity, accessibility, and open-mindedness, enabling a wide range of ideas and contributions (Carmeli et al., 2010). This unique focus on inclusivity is especially relevant in today’s diverse work environments, where leveraging a wide range of talents and perspectives is key to innovation and problem-solving (Nembhard and Edmondson, 2006). Inclusive leadership requires leaders to conduct supportive, open-minded and flexible actions in workplaces, which may inspire employees’ meaning in work (Zhang et al., 2012). However, the specific role and mechanisms of inclusive leadership in the context of career calling have received scant attention. Therefore, this study aims to investigate the association between inclusive leadership and career calling. The research on the relationship between inclusive leadership and career calling not only helps enrich the impact of positive leadership on employees’ sense of meaning, but also makes up for the research gap in the relationship between inclusive leadership and career calling.

The need-to-belong theory posits that individuals have intrinsic motivations to form meaningful connections with others (Baumeister and Leary, 1995). This study will explore how inclusive leadership, which focuses on the connection between individuals and organizational environments, positively relates to individuals’ belongingness and their career calling at work. By integrating these theoretical perspectives, the study aims to provide a comprehensive understanding of the dynamics between inclusive leadership and career calling, offering valuable insights for both academic research and practical leadership development (Baumeister and Leary, 1995). According to Randel et al. (2018), inclusive leadership aims to motivate people to more comprehensively engage in work and offer a chance for all organizational members to release potential abilities. This will encourage employees to have a sense of initiative in establishing high-quality relationships with leaders, in order to view individuals as members of the organization. Therefore, inclusive leadership, characterized by openness, accessibility, and supportiveness, closely relates to a workplace environment that satisfies employees’ need for belongingness. This satisfaction is associated with a stronger sense of identity and integration within the organizational context, with employees also reporting a deeper sense of meaning and purpose in their work, which is related to an enhanced career calling (Xu et al., 2023). This conceptualization aligns with Baumeister and Leary's (1995) assertion that fulfilling the need for belongingness can significantly influence individuals’ emotional, cognitive, and behavioral orientations towards their roles, thus instigating a more profound engagement with their careers as a calling.

One way to improve the career calling of subordinates with belongingness is to have them perceive their own personal values and abilities within the organization. Studies have found that organization-based self-esteem helps to improve employees’ belongingness (Dhir et al., 2024). Influenced by organization-based self-esteem, subordinates with belongingness will achieve results consistent with self-evaluation through improving career calling. Organization-based self-esteem, defined as individuals’ self-perceived value and competence within their organizational role, plays a pivotal role in shaping career calling by influencing how employees perceive their contributions and significance within the workplace (Pierce et al., 1989). High organization-based self-esteem is associated with a positive self-concept in relation to one’s work (Bowling et al., 2010), and this type of employee sees himself as valuable, trustworthy, and contributing to the organization (Pierce et al., 1989). Individuals develop their attitudes in a way that is consistent with their own level of self-esteem (Lee, 2003), so individuals with high organization-based self-esteem may be more likely to report a career calling. This connection is critical for understanding the “black box” of how inclusive leadership is associated with career calling, as it suggests that feeling valued and competent in the organization can lead employees to view their work as a personal calling, beyond mere job satisfaction or role fulfillment. To further elucidate this relationship, it’s essential to explore how inclusive leadership practices that affirm individual contribution and value diversity relate to belongingness based on organization-based self-esteem. This may be associated with employees’ sense of purpose and calling in their careers, as they perceive their work as meaningful and integral to their self-identity.

**Figure 1:**
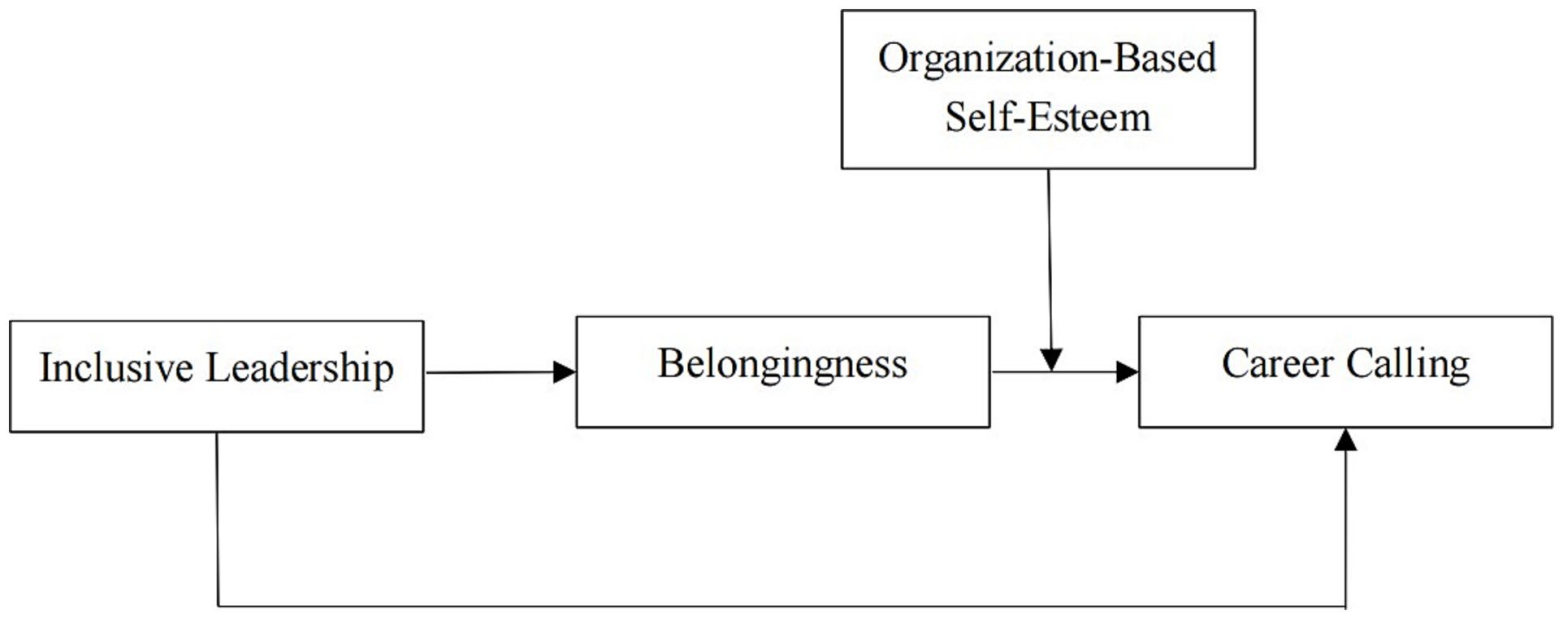
The research model of this paper.

In general, this study will provide some important contributions to the literature on positive leadership and career calling. First, previous research has mainly discussed the outcome variables of career calling, while relatively few studies have been done to discuss which factors relate to career calling. This study expands the scope of influencing factors of career calling via investigating the role of inclusive leadership. Second, this study attempts to use belongingness as a mediator to unlock the “black box” of the two variables. This study fills a critical gap in the literature by exploring how inclusive leadership positively relates to career calling, considering the potential mediating role of belongingness and the moderating role of organization-based self-esteem. While previous research has examined the impact of leadership styles on various outcomes, the specific associations through which inclusive leadership affects career calling have been underexplored (Randel et al., 2018). Third, this study further investigates how the relationship between inclusive leadership and career calling fluctuates under what conditions. Taking organization-based self-esteem as the moderator is conducive to explaining under what conditions the relationship between inclusive leadership and belongingness will be strengthened, and the change of the relationship between inclusive leadership and career calling through belongingness. Empirical evidence supports the notion that inclusive leadership practices—such as demonstrating openness, facilitating accessibility, or being available to others—significantly enhance employees’ belongingness, which in turn positively affects their career calling (Mitchell et al., 2015). Moreover, organization-based self-esteem has been identified as a crucial factor that can amplify the positive effects of inclusive leadership on employees’ belongingness and career calling (Pierce et al., 1989). By addressing these specific relationships, this study enriches the understanding of the relationship between inclusive leadership and career calling, further proposing practical suggestions for organizations seeking to cultivate an inclusive and motivating work environment (Figure 1).

## 2 Theory and hypotheses

### 2.1 The need-to-belong theory

Baumeister and Leary (1995) defined the need for belonging as the basic incentive for people to establish and sustain minimum, long-term lasting, positive and meaningful social connections. Ryan and Deci (2000) extended Baumeister and Leary's (1995) statement and defined the need for belonging as an individual’s need to connect with others, be accepted, respected, and concerned by others, and bring values to others. Accordingly, to meet a person’s need for belonging, one of the most important and basic social motivations of human beings, he/she should experience frequent and pleasant interactions with other people on occasions when people are concerned about each other (Baumeister and Leary, 1995). Furthermore, meeting people’s basic needs may inspire people’s self-determined behaviors (Ryan and Deci, 2000). The positive outcomes of belongingness have been reported by researchers, such as perception of life (Lambert et al., 2013), extra-role behavior (Zhao et al., 2012) and intention to complete goals (Mallet-García and García-Bedolla, 2021). Besides, past literature has also been investigating factors inspiring people’s belongingness and the outcomes. For example, Liu et al. (2022) pointed out that organizational support could increase teleworkers’ belongingness. Furthermore, other social capital factors (e.g., perceived similarity, trust, familiarity) have been verified as predictors of belongingness (Zhao et al., 2012).

This study applies the need-to-belong theory as a theoretical base to investigate the mechanism between inclusive leadership and career calling. Proposed as a kind of supportive leadership, inclusive leadership may inspire employees’ self-determined behaviors by meeting their needs for belongingness. Inclusive leadership can construct a respectful, humane and open climate, which can give living space to different views and beliefs and allow them to be part of organizations. On this occasion, employees’ need for stable and high-quality connections with others may be satisfied (i.e., belongingness). As employees’ basic needs are satisfied, they may recognize organizations and perceive themselves as part of organizations. According to the need-to-belong theory, employees whose needs for belongingness are met may hold positive attitudes toward work. Furthermore, as self-esteem reflects the perceived quality of interpersonal relationships (Baumeister, 2012), it may shape people’s feelings of belongingness and responses to the external environment. Summing up, this study proposes that inclusive leadership can promote satisfaction of people’s belongingness and lead to changes in work attitudes, while being influenced by individuals’ perceived organization-based self-esteem.

### 2.2 Inclusive leadership and career calling

Conceptualized as a broad term for relationship-oriented leadership styles (Korkmaz et al., 2022), inclusive leadership requires leaders to be open, accessible and available in organizational interactions (Carmeli et al., 2010). These requirements shape leaders to conduct altruistic and supportive actions toward subordinates (Černe et al., 2013), such as establishing respectful, responsible and recognizable relations between supervisors and subordinates (Hollander, 2009), listening and concerning subordinates’ needs and expectations (Carmeli et al., 2010), and respecting diversity and divergence in groups (Nembhard and Edmondson, 2006). Past literature has confirmed the essential role of inclusive leadership in predicting performance. In terms of individual level, inclusive leadership has been verified to contribute to positive subordinates’ outcomes, such as motivation (Zhang and Jin, 2019), working performance (Khan et al., 2020), extra-role behavior (Jiang et al., 2020), and in-role behavior (Chen et al., 2020). Furthermore, the positive effects of inclusive leadership can be found at team and organizational levels in various aspects, including performance (Qi and Liu, 2017), climate (Li and Peng, 2022) and team-level identity (Mitchell et al., 2015). Although organizations believe that inclusive leadership may ensure micro and meso-level performance, the high turnover rate among young employees in organizations due to the pandemic and financial crises break their assumptions (Deal et al., 2010; Ng et al., 2010). Employees have turnover intention due to a lack of sense of work meaning.

Defined as strong enthusiasm and meaningful passion toward work perceived by individuals (Dobrow and Tosti-Kharas, 2011), career calling consists of three dimensions, including prosocial orientation, transcendent summons and purposeful work dimensions (Dik et al., 2012). Past literature has reported various benefits of career calling to organizations, such as career commitment (Lv et al., 2021), meaning in life (Peng et al., 2020) and low burnout (Zhao et al., 2022). Given the positive effects of career calling in organizations and the potential contribution of leadership to shaping employees’ cognitions and motivations (Gupta, 2020), scholars in leadership conversation have started to devote themselves to it. For instance, Zhang and Jiang (2020) found that ethical leadership could inspire Chinese employees’ career calling. However, the research about inclusive leadership and career calling is scarce.

As a kind of relation-focused leadership, inclusive leadership emphasizes the interactions between supervisors and subordinates (Randel et al., 2018). Inclusive leaders interact with employees and influence their career calling through openness, accessibility and availability (Carmeli et al., 2010). First, open and accessible inclusive leaders have flexibility and curiosity. They respect each subordinate, accept employees’ different new views and ideas, and encourage employees to participate in decision-making (Darvishmotevali, 2019). This way of being recognized by leaders will enhance employees’ sense of ownership and responsibility, improve employees’ perception of the meaning of their work (Brown and Mitchell, 2010), and inspire employees’ career calling. The support of inclusive leadership is conducive to the improvement of personal ability (Carmeli et al., 2010), and the higher the matching degree between personal ability and job requirements, the stronger the career calling (Thompson and Bunderson, 2019). Finally, accessible and inclusive leaders usually pay attention to the needs and expectations of subordinates and provide help to subordinates in a timely manner. When employees are respected and authorized by leaders (Hollander, 2009), they can freely and equally express their suggestions and ideas in the process of participating in enterprise decision-making (Nishii, 2013). The positive attitudes and expectations toward the job may make them achieve beyond themselves and realize the meaning of work. Based on the above narration, the study posits that:

***H1**: The relationship between inclusive leadership and subordinates’ career calling is positive.*

### 2.3 Inclusive leadership and belongingness

Inclusive leadership promotes employees’ belongingness in three ways: supporting team members, ensuring fairness in experience for each member, and allowing participation in shared decision-making toward organizations’ issues (Randel et al., 2018). First, during the interactions among colleagues, inclusive leaders value the concern, care toward subordinates in the daily work, patiently listen to communications, free expression, and respect and support for subordinates’ needs (Hollander, 2009). In this case, subordinates may have a sense of being accepted, recognized and respected by the organizations (Won et al., 2018) and perceive the fairness during the interactions. These good senses and perceptions toward interactions will make subordinates regard themselves as part of organizations and satisfy subordinates’ need of belongingness (Baumeister and Leary, 1995). Second, inclusive leaders demonstrate to group members that they are a respected part of the group by treating them in a fair and just manner (Sabharwal, 2014; Shore et al., 2011). Third, inclusive leadership shares decision-making power with subordinates by inviting employees to participate and make decisions together, expands decision-making consultation, and helps subordinates decide how to work. This promotes the belongingness of subordinates (Nembhard and Edmondson, 2006).

***H2**: The relationship between inclusive leadership and subordinates’ belongingness is positive.*

### 2.4 Belongingness as a mediator

In light of the need-to-belong theory, this study posits that the establishment and maintenance of interpersonal relationships are not just fundamental to human activity but are also pivotal in shaping one’s career calling (Pickett et al., 2004). Belongingness, conceptualized as the desire and fulfillment of being an integral part of a group or organization, emerges as a critical psychological state that mediates the relationship between inclusive leadership and career calling. Inclusive leadership, characterized by its emphasis on fostering a supportive, open, and respectful environment, enhances employees’ belongingness by making them feel valued, respected, and an essential part of the organizational fabric (Randel et al., 2018). Furthermore, people’s behaviors may be motivated by the extent of being recognized as part of groups and taken care of by others (Ryan and Deci, 2000). For example, Hoogervorst et al. (2012) and Dysvik et al. (2013) found that the more leaders and followers perceived belongingness, the more they might conduct altruistic behaviors. Besides, scholars have confirmed the positive effect of belongingness on employees’ psychology. Shakespeare-Finch and Daley (2017) revealed that workplace belongingness could control distress and inspire resilience. Although scholars have found different benefits of belongingness in organizations, they still need more work on the fostering factors. Responding to Randel et al.'s (2018) appeal to introducing leaders to inspiring belongingness, Kyei-Poku and Yang (2020) found that authentic leadership could indirectly inspire followers’ organizational citizen behaviors via belongingness.

This amplified belongingness, in turn, has profound implications for employees’ career calling. It deepens their connection with their work, instilling it with heightened meaning, passion, and a transcendental quality. This transcends the conventional parameters of job satisfaction or engagement, positioning career calling as a profound alignment with one’s professional role, reflective of personal values and purpose. This study, therefore, posits that belongingness mediates the relationship between inclusive leadership and career calling, translating inclusive practices into a more pronounced career calling among employees. Kyei-Poku (2014) further underscores this relationship by illustrating how perceived belongingness can inspire altruistic behaviors and a stronger commitment to organizational goals, aspects closely related to the concept of career calling. The sense of security and identity derived from belongingness motivate employees to contribute meaningfully to their organizations, enhancing their sense of purpose and fulfillment from their work. This study hypothesizes:

***H3**: Belongingness positively mediates the relationship between inclusive leadership and career calling.*

### 2.5 Moderating effects of organization-based self-esteem

Defined as the perceived value of individuals as organizational members in the organizational environment (Pierce et al., 1989), self-esteem was one of the main factors inspiring individuals’ psychological well-being and meaning in life (Lee and Peccei, 2007). Later, Pierce and his/her colleagues proposed the concept of organizational self-esteem and clarified its definition as the degree to which individuals perceive their own competence, importance, value, respect, and match with the organization rather than as individuals’ perceptions of their own value in the organizations and the extent to which they are capable of participating in organizational actions to the degree to which individuals perceive their own competence, importance, value, respect, and match with the organization (Pierce et al., 1989; Pierce and Gardner, 2004). Past literature has revealed the effect of organization-based self-esteem in shaping individuals’ behaviors. According to Brockner (1988), people with low levels of organization-based self-esteem will have comparatively pessimistic attitudes and be sensitive to negative stimulations. Pierce and Gardner (2004) also argued that researchers might consider the moderating effect of organization-based self-esteem in the working environment and individuals’ behaviors.

Need-to-belong theory reflects that human’s basic need for belongingness has a strong impact on emotions, cognitions and behaviors (Baumeister and Leary, 1995). In this study, subordinates with high-level organization-based self-esteem are sensitive to external positive stimulations (Brockner, 1988) and believe that individuals are valuable, trustworthy and contributive to the organization (Gardner and Pierce, 1998). When these subordinates keenly notice the feeling of being part of the organizations, they may feel confident, which can make employees with a high belongingness believe that individuals have good interpersonal relationships in the organization. They will actively explore the meaning and value of work, give full play to their potential, and generate a high career calling through strong internal motivation (Dumulescu et al., 2015). At the same time, subordinates with high organization-based self-esteem may have positive attitudes toward organizations because they easily feel support from groups (Korman, 1976). In this case, they believe that individuals in the groups are important to them, which motivates these subordinates to altruistically help others. Accordingly, this study proposes that:

***H4**: Organization-based self-esteem moderates the relationship between belongingness and career calling, such that this relationship is stronger when the organization-based self-esteem is high than when it is low.*

### 2.6 Moderated mediating effect

In a similar way, the mediating effect of belongingness is also influenced by organization-based self-esteem. When subordinates have high-level organization-based self-esteem, they may believe that individuals can contribute to the organizations and participate in organizations’ work and activities, which is essential and meaningful for organizational development (Kim and Beehr, 2018). In an inclusive leadership context, subordinates may perceive the leaders’ recognition, encouragement and support better, which may be accompanied by their belongingness. On this occasion, subordinates may be more passionate about their work, brave enough to accept challenges, diligent enough to achieve purposes, and willing to help other members because they may believe that the organizations are vital and their work is valuable. On the contrary, subordinates with low-level organization-based self-esteem will have lower perceptions of self-competence, hardly perceive support from organizations, and hold skeptical attitudes toward whether they are accepted by the organizations or not, which may lead them to feel a sense of incapability and not easy to experience the work passion and sense of meaning. In accordance with the above, the study posits that:

***H5**: Organization-based self-esteem positively moderates the indirect relationship between inclusive leadership and career calling via belongingness, such that this indirect relationship is stronger when the organization-based self-esteem is high than when it is low.*

## 3 Research methods

### 3.1 Research subjects and collection procedures

This study regarded Chinese employees as the population and recruited participants from different firms in various industries in Guangdong, Zhejiang, and Beijing provinces, China. This study applied a snowball sampling to contact 11 managers in the human resources management departments of 11 companies to ask for support in accessing employees and collecting data. After gaining permission from companies, researchers adopted a convenient sampling approach to recruit 500 potential participants. To control common method bias, we applied a time-lagging approach to collect data over two periods (Podsakoff et al., 2003). The interval between the two collections is 2 months. Before the data collection, the study sent consent forms, participation information sheets, and hard-copied questionnaires to participants and ensured that their participation was voluntary and confidential. In wave 1, 500 surveys were distributed to participants to respond to questions about inclusive leadership and belongingness. Furthermore, researchers asked participants to report their personal information (e.g., gender, age, phone numbers and email address). In wave 1, the study received 420 responses and numbered these surveys. Two months later, the study invited participants who were matched to respond to questions related to organization-based self-esteem and career calling. In wave 2, the study gained 407 surveys. Finally, the study received 337 surveys after deleting the invalid ones (those failing to answer all questions), accounting for 67.4% of the initial number of participants. Table 1 described the demographic characteristics of the participants. Of these, a third of participants are from other industries, namely technology, medical and transportation.

**Table 1:** Descriptive characteristics of the sample.

Characteristic	Percent (Number)
Gender	
Male	48.1% (162)
Female	51.9% (175)
Age (years)	
<20	26.1% (88)
21–30	36.2% (122)
31–40	27.3% (92)
41–50	9.2% (31)
>50	1.2% (4)
Marital status	
Non-married	53.4% (180)
Married	42.7% (144)
Divorced	3.9% (13)
Education level	
Doctor’s degrees	4.2% (14)
Master’s degrees	7.1% (24)
Bachelor’s degrees holders	51.3% (173)
Junior college diplomas	19% (64)
Technical secondary school, High school or below	18.4% (62)
Tenure (years)	
<10	94.7% (319)
> = 10	5.3% (16)
Time working with a fixed leader (years)	
<10	98.5% (332)
> = 10	1.5% (5)
Industry	
Educational	36.2% (122)
Financial	3.3% (11)
Service	23.1% (78)
Construction	3.9% (13)
Other (Technology, Medical, Transportation)	33.5% (113)

### 3.2 Ethics approval and consent to participate

All participants in this paper have provided written informed consent in order to collect information and publish the data generated by the article study and its results. This project adopts questionnaires to collect data and is approved by the Institutional Review Committee of Yiwu Industrial and Commercial College. Sign and submit to the “Ethical Issues Form” provided by Yiwu Industrial and Commercial College to ensure the authenticity of this paper and conform to academic ethics. In addition, the study was conducted in accordance with the Declaration of Helsinki.

### 3.3 Measuring tools

Following Brislin’s (1980) suggestions, this study adopted several 5-point Likert scales from the existing literature (1 refers to strongly disagree attitude, 5 refers to strongly agree attitude) and translated the scales into Chinese versions. The study firstly invited two overseas researchers with human resources management backgrounds to translate the English versions into Chinese versions. Then, the research team invited two professors in related fields to translate the Chinese versions back to English versions and assisted the research team in modifying the Chinese versions based on the cultural context into final versions.

#### 3.3.1 Inclusive leadership

Participants completed a nine-item scale developed by Carmeli et al. (2010) to measure inclusive leadership. The scale included three dimensions, including 3 items about openness (e.g., The manager is open to hearing new ideas), 2 items about accessibility (e.g., The manager encourages me to access him/her on emerging issues) and 4 items about availability (e.g., The manager is available for consultation on problems). The Cronbach’s alpha reaches a satisfying level (0.947).

#### 3.3.2 Belongingness

A 12-item scale developed by Malone et al. (2012) was used to ask participants to report their attitudes toward belongingness. Among the 12 items, items 3, 4, 6, 7, 9 and 12 were reverse-scored (e.g., I feel isolated from the rest of the world). The Cronbach’s alpha reaches a satisfying level (0.921).

#### 3.3.3 Organization-based self-esteem

This study applied a widely adopted scale developed by Pierce et al. (1989) to measure organization-based self-esteem. The scale had 10 items and showed satisfying reliability in this study. An example item was “I am trusted.” The Cronbach’s alpha reaches a satisfying level (0.945).

#### 3.3.4 Career calling

The study applied the 12-item scale developed by Dobrow and Tosti-Kharas (2011) to measure career calling. The example item was “I am passionate about being an employee in the company.” The Cronbach’s alpha reaches a satisfying level (0.959).

#### 3.3.5 Control variables

Based on previous studies, gender, age, and tenure have been found to influence individual career calling (Chen et al., 2018; Creed et al., 2016; Wu and Lee, 2020). To more accurately validate the model, gender (male = 0, female = 1), age (year), and tenure (year) were measured as control variables.

### 3.4 Methods for data analysis

Data analyses can be divided into several steps. First, we test the validity of the measurement model through confirmatory factor analysis by applying AMOS. We calculated overall scores for all items on all dimensions of each variable (inclusive leadership, occupational calling, belonging, organization-based self-esteem). Then, we computed the mean score for each variable by averaging these overall scores. We carried out descriptive statistics and correlation analysis to conduct preliminary data analysis. Later, we used SPSS and PROCESS to test the hypotheses (Edwards and Lambert, 2007; Hayes, 2017; Preacher and Hayes, 2004). The data analysis for this study was conducted in a series of steps to rigorously test the hypotheses and explore the relationships between inclusive leadership, belongingness, organization-based self-esteem, career calling, and control variables (i.e., age, gender, and tenure in this study).

We tested our hypothesis through multiple linear regression analysis in SPSS and Process macro. Multiple regression analysis is widely used to test models in management (Montani et al., 2020; Pan et al., 2018). To examine the mediating role of belongingness (BE) between inclusive leadership (IL) and career calling, we utilized the PROCESS macro in SPSS, following Preacher and Hayes's (2004) bootstrapping method. In examining the moderating effect, since regression analyses involve the interaction between belongingness and organization-based self-esteem (OBSE), we referred to Aiken and West (1991) and centralized values of belongingness and organization-based self-esteem to avoid exaggerating in multicollinearity, seeking a better interpretation.

## 4 Research results

### 4.1 Common method bias test

As the data was self-reported by participants, there might exist common method bias. Thus, this study applied two ways to test the common method bias. The study applied a Harman’s single-factor test to diagnose the common method bias. The result showed that common method bias was not a serious issue, as a single factor accounted for 39.233% of the variance (Podsakoff et al., 2003).

### 4.2 Confirmatory factor analysis

To test the validity of scales from existing literature, we used confirmatory factor analysis to test the measurement model via AMOS software (Table 2). Compared with other alternative models, the four-factor model satisfyingly fitted the data. According to Medsker et al. (1994), all the results of indexes met the cut-point of criteria (*χ*^2^ = 1620.140, *df* = 854, RMSEA = 0.052, SRMR = 0.056, CFI = 0.933, TLI = 0.929, IFI = 0.933). Thus, the validity of the measurement model was ensured. According to the approach (ULMC) suggested by Bagozzi and Yi (1988) of testing common method variance (CFI = 0.950, TLI = 0.947, RMSEA = 0.045, SRMR = 0.048), there is no serious common method variance (∆CFI = 0.017, ∆TLI = 0.018, ∆RMSEA = 0.007, ∆SRMR = 0.008).

**Table 2:** Results of confirmatory factor analysis (*N* = 337).

Models	*χ* ^2^	*df*	RMSEA	SRMR	CFI	TLI
Four-factor model (hypothesis)	1620.140	854	0.052	0.056	0.933	0.929
Three-factor model (A + B)	2936.302	857	0.085	0.094	0.818	0.808
Three-factor model (A + C)	3179.278	857	0.090	0.099	0.796	0.785
Three-factor model (A + D)	3200.472	857	0.090	0.093	0.794	0.783
Two-factor model (A + B, C + D)	5320.227	859	0.124	0.164	0.608	0.588
One-factor model (A + B + C + D)	6258.182	860	0.137	0.145	0.526	0.503

A (Inclusive leadership), B (Belongingness), C (Career calling), D (Organization-based self-esteem).

### 4.3 Correlation analysis

Before testing the hypotheses, this study conducted descriptive statistics and correlation analyses to draw a simple picture of the data. Table 3 shows descriptive statistics (means and standard deviations) and correlation coefficient among variables. The descriptive statistics showed that the participants’ attitudes toward variables were generally high as their mean scores of variables were located in more than 3.6. Furthermore, though the standard deviation of belongingness was larger than that of other variables, the dispersion degrees of variables were generally similar. Besides, given that the Kolmogorov–Smirnov normality test showed that all the data were non-normally distributed (*p*-value <0.5), Spearman’s correlation analysis was applied to investigate the variables’ relationships. Table 2 showed that inclusive leadership was positively correlated with belongingness (*r* = 0.447, *p* < 0.01) and career calling (*r* = 0.573, *p* < 0.01), respectively. Subsequently, belongingness (*r* = 0.488, *p* < 0.01) and organization-based self-esteem (*r* = 0.313, *p* < 0.01) were positively correlated with career calling, respectively.

**Table 3:** Descriptive statistics (means and standard deviations) and correlation analyses among variables (*N* = 337).

Variables	Mean	SD	1	2	3	4	5	6
1.Gender	–	–						
2.Age	28.85	8.539	−0.349**					
3.Tenure	–	–	−0.230**	0.387**				
4.IL	4.144	0.777	−0.039	0.088	−0.019			
5.CC	3.983	0.756	0.035	−0.132*	−0.097	0.589**		
6. BE	3.652	0.829	0.008	−0.171**	−0.069	0.454**	0.516**	
7.OBSE	4.003	0.737	0.020	0.066	0.089	0.548**	0.293**	0.342**

***p < 0.001, **p < 0.01, *p < 0.05; IL (Inclusive Leadership), BE (Belongingness), CC (Career Calling), OBSE (Organization-Based Self-Esteem).

### 4.4 Hypotheses testing

#### 4.4.1 Main effect test

The hypothesis testing in this study was methodically structured and executed through a series of analytical steps using SPSS 23 and the PROCESS macro, as recommended by Preacher and Hayes (2004). First, we use SPSS software to assess the main effect of inclusive leadership on career calling (Hypothesis 1). As shown in Model 1 in Table 4, the results show a positive correlation (b = 0.59, *p* < 0.001), thus supporting H1.

**Table 4:** Results of regression analysis.

variable	BE		CC					
	Model1	Model 2	Model 3	Model 4	Model 5	Model 6	Model 7	Model 8
Gender	−0.100 (−0.061, 0.096, −1.046)	−0.087 (−0.053, 0.084, −1.038)	−0.028 (−0.019, 0.008, −0.323)	−0.013 (−0.009, 0.070, −0.193)	0.018 (0.012, 0.076, 0.239)	0.009 (0.006, 0.067, 0.134)	0.009 (0.006, 0.075, 0.114)	0.025 (0.016, 0.066, 0.376)
Age	−0.018** (−0.188, 0.006, −3.083)	−0.023*** (−0.239, 0.005, −4.436)	−0.010 (−0.117, 0.005, −1.898)	−0.016*** (−0.182, 0.004, −3.696)	−0.002 (−0.021, 0.005, 0.386)	−0.010* (−0.114, 0.004, −2.374)	−0.004 (−0.049, 0.005, −0.923)	−0.011* (−0.122, 0.004, −2.555)
Tenure	−0.002 (−0.010, 0.013, −0.175)	0.004 (0.020, 0.011, 0.395)	−0.011 (−0.057, 0.012, −0.957)	−0.003 (−0.017, 0.009, −0.37)	−0.010 (−0.051, 0.010, −1.005)	−0.005 (−0.023, 0.009, −0.516)	−0.012 (−0.059, 0.010, −1.168)	−0.002 (−0.010, 0.009, −0.219)
IL		0.505*** (0.474, 0.051, 9.94)		0.588*** (0.064, 0.042, 13.909)		0.458*** (0.471, 0.046, 5.901)		0.493*** (0.507, 0.045, 9.636)
BE					0.464*** (0.509, 0.043, 10.686)	0.257*** (0.281, 0.043, 9.991)	0.384*** (0.420, 0.384, 8.059)	0.236*** (0.258, 0.045, 5.257)
OBSE							0.191** (0.187, 0.054, 3.564)	−0.038 (−0.037, 0.053, −0.711)
IL*OBSE							0.143* (0.123, 0.058, 2.485)	0.137** (0.117, 0.051, 2.688)
*R^2^*	0.032	0.254	0.020	381	0.271	0.440	0.302	0.456
*△R^2^*		0.222***		0.361***	0.251***	0.059***	0.031**	0.016**
*df1/df2*	3/333	1/332	3/333	1/332	1/332	5/331	2/330	2/329
*F(p)*	3.725 (0.012)	28.314 (0.000)	2.295 (0.078)	51.081 (0.000)	30.851 (0.000)	51.993 (0.000)	23.821 (0.000)	39.366 (0.000)

IL (Inclusive Leadership), BE (Belongingness), CC (Career Calling), OBSE (Organization-Based Self-Esteem). b (β, standard error, t): unstandardized coefficients (b), standardized coefficients (β), *t*-test(*t*). *N* = 337, *p < 0.05, **p < 0.01, ***p < 0.001.

#### 4.4.2 Test for the mediating effect

Subsequently, we examined the mediating impact of belongingness in the main path by using SPSS software for regression analysis (Hypothesis 2). In Model 4 and Model 6, as shown in Table 5, the data results show that the direct effect of inclusive leadership on career calling decreases from b = 0.59 (*p* < 0.001) to b = 0.46 (*p* < 0.001) when belongingness is taken as the mediating variable. There was a strong positive correlation between belongingness and career calling (b = 0.26, *p* < 0.001). Although the direct relationship between inclusive leadership and career calling was reduced, it also remained significant. This indicates that belongingness partially mediates the relationship between inclusive leadership and career calling. In addition, in order to further examine the mediating role of belongingness, this study combined with the Process program. The data results are shown in Table 5 (Effect = 0.13, se = 0.03, 95%CI = [0.07, 0.19]), which also proves Hypothesis 2.

**Table 5:** Mediation effect and moderated mediation effect.

	Indirect effect	SE	95%CI
Simple mediation effect			
	0.130	0.029	[0.073, 0.185]
Moderated mediation effect			
High moderator (OBSE)	0.170	0.035	[0.102, 0.234]
Mean (OBSE)	0.119	0.025	[0.071, 0.167]
Low moderator (OBSE)	0.068	0.029	[0.008, 0.124]

Bootstrap sample size *N* = 5,000; OBSE (Organization-Based Self-Esteem).

#### 4.4.3 Testing belongingness as a moderator

**Figure 2:**
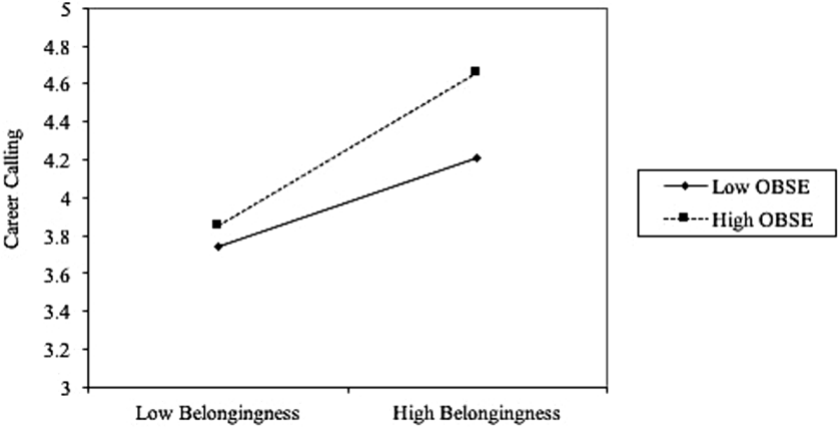
Moderating effect of organization-based self-esteem.

**Figure 3:**
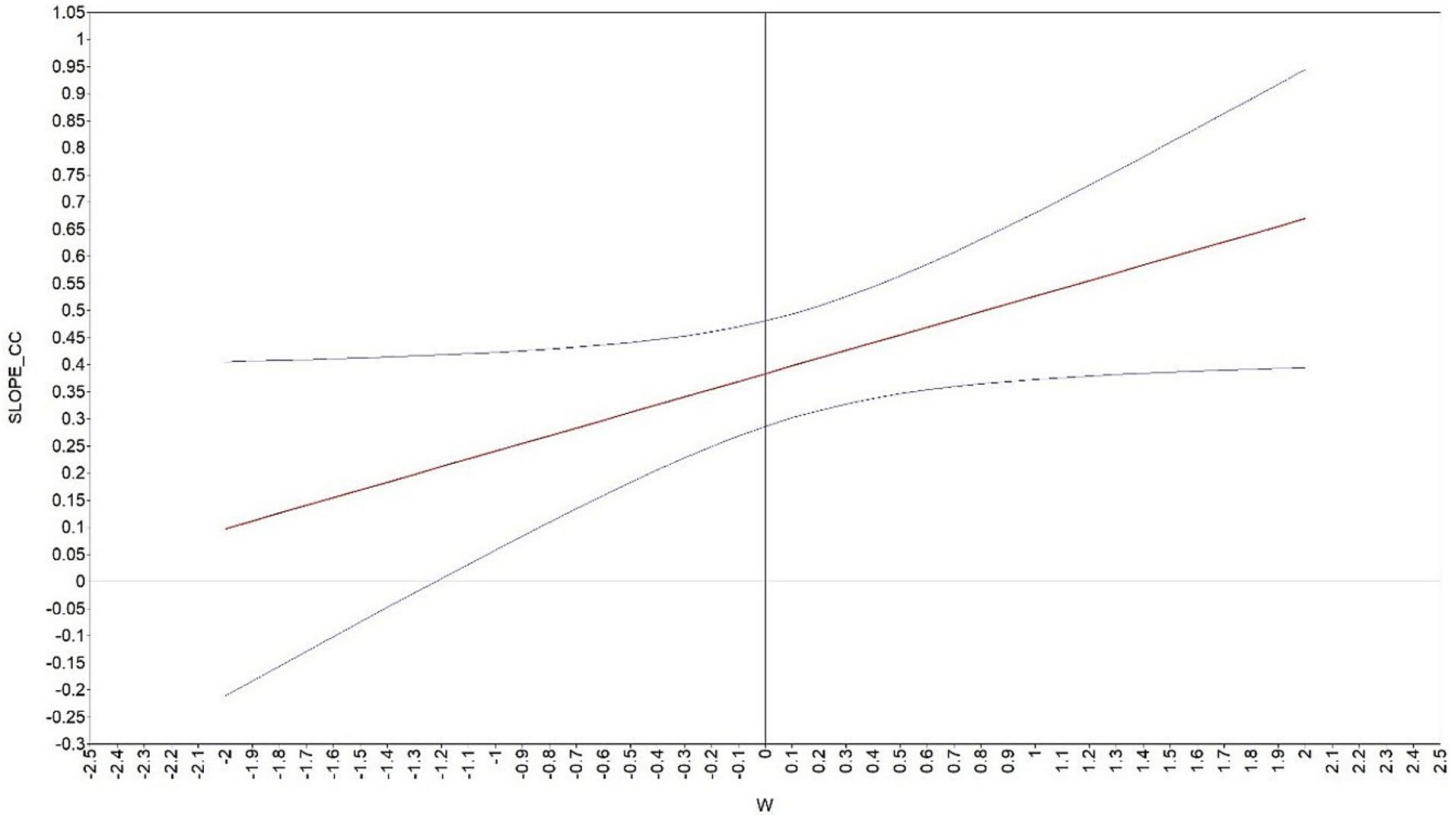
Organization-based self-esteem (J-N) as a moderator.

For the moderating effect analysis (Hypothesis 3), the interaction term was calculated by multiplying belongingness with organization-based self-esteem. We used centralized belongingness and organization-based self-esteem to control multicollinearity and seek a better interpretation (Aiken and West, 1991). As shown in Model 7 in Table 4, based on the Model 5, the centralized belongingness, organization-based self-esteem and their interaction terms are added as independent variables. The analysis revealed that both belongingness (b = 0.38, *p* < 0.001) and organization-based self-esteem (b = 0.19, *p* < 0.01) were positively associated with career calling, and with the interaction term also showing a significant positive effect (b = 0.14, *p* < 0.05). Hypothesis 3 was proved. Figure 2 more intuitively shows the moderating effects of organization-based self-esteem. We also used the method suggested by McCabe et al. (2018) to enhance the visualization of the adjustment renderings through the J-N diagram. Since the interval shown on the graph does not contain 0, the moderated effect is valid (Figure 3). Hypothesis 3 is supported.

#### 4.4.4 Moderated mediating effect test

We applied multiple steps to validate Hypothesis 4. We first used SPSS software for regression analysis. Based on Model 6, Model 8 in Table 4 added centralized organization-based self-esteem, interaction terms of organization-based self-esteem and belongingness as independent variables to regression the career calling. The data results showed that (Model 8 in Table 4), inclusive leadership (b = 0.49, *p* < 0.001), belongingness (b = 0.24, *p* < 0.001), and the interaction coefficient (b = 0.14, *p* < 0.01) were significant. In order to further test the moderating effect of organization-based self-esteem, model 14 of PROCESS was adopted. Table 5 exhibits that this effect was stronger at a higher level of organization-based self-esteem (Boot indirect effect = 0.17, Boot SE = 0.035, 95%CI = [0.10, 0.23]), supporting Hypothesis 4.

## 5 Discussions

In this study, we explored the relationship between inclusive leadership and career calling, particularly focusing on the mediating role of belongingness and the moderating role of organization-based self-esteem. Our findings not only corroborate existing research on the positive associations between inclusive leadership and various employee outcomes but also extend the literature by elucidating the psychological mechanisms underpinning these relationships. The results suggest that inclusive leadership is associated with higher levels of employees’ career calling. Subsequently, belongingness mediates the relationship between inclusive leadership and career calling. Furthermore, organization-based self-esteem not only positively moderates the relationship between belongingness and career calling but also moderates the indirect path between inclusive leadership and career calling via belongingness.

### 5.1 Theoretical implications

This study has some theoretical contributions. First, this study contributes to career calling literature by extending the positive relationship between inclusive leadership and career calling. Consistent with past research, positive leadership styles are beneficial for improving employees’ career calling, for example, meaningful leadership (Ahmad and Fatima, 2023), authentic leadership (Liu and Wong, 2023), caring leadership (Zhang et al., 2024) and empowering leadership (Zhang and Jin, 2019). However, limited scholars have focused on the relationship between inclusive leadership and employees’ career calling. This study tests this relationship, enriching the research on the factors that influence career calling.

Second, this study reveals the relationship mechanism between inclusive leadership and career calling by introducing a mediating variable (belongingness). It responds to the call for more evidence on the belongingness in workplaces (Cockshaw et al., 2013) and the nature of belongingness in inclusive leadership (Randel et al., 2018). Inclusive leadership as a positive style of leadership is consistent with past research that positive leadership improves employees’ belongingness to the organization and team, for example, appreciation leadership (Saleh et al., 2024) and charismatic leadership (Den Hartog et al., 2007). By recognizing the applicability of the need for belonging theory in inclusive leadership, this study extends the conclusion of Katsaros (2022) by revealing inclusive leadership can satisfy employees’ need for belongingness and directly inspire employees’ positive attitudes and behaviours (i.e., career calling in this study) via providing supportive and fair treatment and climates in Eastern context.

Third, this study contributes to the study of boundary conditions of the relationship between inclusive leadership and belongingness. The results of this study also confirm the moderating role of organization-based self-esteem (Rank et al., 2009; Zhao and Liu, 2022) and the positive impact of organization-based self-esteem on belongingness (Carnevale et al., 2018; Dhir et al., 2024; Ferris et al., 2009). Based on past research, filling the gap in neglecting the moderating effect of organization-based self-esteem in the effectiveness of inclusive leadership literature, this study indicated that positive associations between inclusive leadership and employees’ belongingness, as well as between inclusive leadership and career calling, varied among employees with different levels of organization-based self-esteem. These findings indirectly supported the idea that self-esteem reflected the quality of relationships in belongingness theory. Besides, the verification of the moderating effect of organization-based self-esteem extends the role of organization-based self-esteem from shaping individual actions (Su et al., 2021) or varying individuals’ cognitions (Kuo and Wu, 2022) to results of interactions between external environmental factors (e.g., leadership) and individual perception (e.g., belongingness), which can encourage researcher’s further exploration of the scope of organization-based self-esteem.

### 5.2 Practical implications

We underscore the significant role of inclusive leadership in cultivating an environment that is closely linked with employees’ career calling. Inclusive leadership, characterized by openness, accessibility, and affirmation, has been empirically linked to enhanced employee engagement and satisfaction (Mitchell et al., 2015). Managers should, therefore, prioritize creating an inclusive atmosphere where diverse perspectives are valued and mistakes are seen as learning opportunities. Instead of imposing harsh penalties for errors, a more constructive approach involves providing constructive feedback and support to help employees learn and grow from their experiences. This way of managing people resonates with the principles of inclusive leadership but also promotes a culture of continuous improvement and resilience (Liu et al., 2022).

Further, the mediating role of belongingness in the relationship between inclusive leadership and career calling suggests that fostering a strong sense of community within the organization is pivotal. Empirical studies have shown that team-building activities, such as regular team dinners and informal gatherings, can significantly link to employees’ sense of belongingness (Hoogervorst et al., 2012). These activities provide informal platforms for employees to connect, share experiences, and reinforce their commitment to the organization’s goals, which may be linked with their belongingness. Regarding organization-based self-esteem, the study highlights its moderating role when career calling appears in the inclusive leadership context. Systematic and frequent training programs aimed at skill development and personal growth have been shown to enhance employees’ self-esteem and perceived competence within the organization (Ryan and Deci, 2000). By investing in employees’ development, organizations can not only improve self-esteem but also foster a culture of empowerment and autonomy, leading to more effective leadership and improved organizational outcomes.

In conclusion, the practical implications of this study suggest that inclusive leadership practices, coupled with targeted activities to enhance belongingness and organization-based self-esteem, can significantly relate to a supportive work environment that is closely linked to career calling. By implementing these strategies, organizations can retain talented employees, maintain their enthusiasm, and enhance their overall productivity and satisfaction.

### 5.3 Limitations and future research directions

Although this study makes contributions to the literature to some extent, some limitations need to be addressed for future research. First, given the cross-sectional research design, the findings cannot deduce causal inference. To predict the effectiveness of leadership and the dynamic changes in people’s attitudes and beliefs, researchers may conduct longitudinal studies or experiments to test the conclusion proposed in this study.

Second, the data in this study are from Chinese employees, which cannot represent all people’s beliefs and attitudes in China and the world. To generalize the conclusions from the study, other researchers may try to collect data from samples from different nations or areas to retest the model.

Third, the study collects data only from employees, which makes data biased because single source data (i.e., self-report survey) cannot reflect all aspects of leadership or career calling phenomena and thoughts of participants. Thus, researchers in the same conversation may try to test the hypothesis with multi-source data (e.g., online comments, peer assessment, superior assessment) to understand related phenomena and investigate their relationships.

Finally, the mechanism existing in the inclusive leadership-career calling link is complex. Hence, more studies may try to investigate the roles of organization-level and team-level factors in the link or the combined effects of different types of factors in shaping the relationship between inclusive leadership and career calling.

## 6 Conclusion

Although scholars have focused on investigating the employees’ career calling in the leadership context, further research is needed on the potential pathways through which inclusive leadership may be related to career calling. Based on the Need-to-belong theory, we collect data from Chinese employees through self-reported surveys and verify belongingness as a mediator and organization-based self-esteem as a moderator in the inclusive leadership-career calling path, which contributes to the career calling literature. Besides, the study proposes several implications for practitioners in the Chinese business context and research avenues for future research.

## Data Availability

The raw data supporting the conclusions of this article will be made available by the authors, without undue reservation.
